# The carbohydrate at asparagine 386 on HIV-1 gp120 is not essential for protein folding and function but is involved in immune evasion

**DOI:** 10.1186/1742-4690-5-10

**Published:** 2008-01-31

**Authors:** Rogier W Sanders, Eelco van Anken, Alexei A Nabatov, I Marije Liscaljet, Ilja Bontjer, Dirk Eggink, Mark Melchers, Els Busser, Martijn M Dankers, Fedde Groot, Ineke Braakman, Ben Berkhout, William A Paxton

**Affiliations:** 1 Laboratory of Experimental Virology, Dept. Medical Microbiology, Center of Infection and Immunity Amsterdam (CINIMA), Academic Medical Center of the University of Amsterdam, Amsterdam, The Netherlands; 2 Cellular Protein Chemistry, Bijvoet Center for Biomolecular Research, Utrecht University, Padualaan 8, 3584 CH Utrecht, The Netherlands; 3 Department of Biochemistry and Biophysics, University of California, San Francisco, CA 94158-2517, USA; 4 Department of Molecular Cell Biology and Immunology, VU University Medical Center, van de Boechorstraat 7, 1081 BT Amsterdam, The Netherlands; 5 Crucell, Archimedesweg 4, 2333 CN Leiden, The Netherlands; 6 Sir William Dunn School of Pathology, University of Oxford, South Parks Road, Oxford OX1 3RE, UK

## Abstract

**Background:**

The HIV-1 envelope glycoprotein gp120, which mediates viral attachment to target cells, consists for ~50% of sugar, but the role of the individual sugar chains in various aspects of gp120 folding and function is poorly understood. Here we studied the role of the carbohydrate at position 386. We identified a virus variant that had lost the 386 glycan in an evolution study of a mutant virus lacking the disulfide bond at the base of the V4 domain.

**Results:**

The 386 carbohydrate was not essential for folding of *wt *gp120. However, its removal improved folding of a gp120 variant lacking the 385–418 disulfide bond, suggesting that it plays an auxiliary role in protein folding in the presence of this disulfide bond. The 386 carbohydrate was not critical for gp120 binding to dendritic cells (DC) and DC-mediated HIV-1 transmission to T cells. In accordance with previous reports, we found that N386 was involved in binding of the mannose-dependent neutralizing antibody 2G12. Interestingly, in the presence of specific substitutions elsewhere in gp120, removal of N386 did not result in abrogation of 2G12 binding, implying that the contribution of N386 is context dependent. Neutralization by soluble CD4 and the neutralizing CD4 binding site (CD4BS) antibody b12 was significantly enhanced in the absence of the 386 sugar, indicating that this glycan protects the CD4BS against antibodies.

**Conclusion:**

The carbohydrate at position 386 is not essential for protein folding and function, but is involved in the protection of the CD4BS from antibodies. Removal of this sugar in the context of trimeric Env immunogens may therefore improve the elicitation of neutralizing CD4BS antibodies.

## Background

The HIV-1 envelope (Env) glycoproteins (gp120 and gp41) mediate viral entry into target cells by binding to the appropriate cellular receptors and facilitating fusion of viral and cellular membranes. The ectodomain of the Env complex is composed for ~50% of carbohydrates that have multiple functions. i) Proper folding of Env in the Endoplasmic Reticulum (ER) is dependent on glycosylation and Env misfolding occurs in the presence of glycosylation inhibitors [1-3]. ii) Carbohydrate moieties are important for HIV-1 binding to C-type lectins on dendritic cells (DCs), such as DC-SIGN, which have been implicated in early viral transmission events and dissemination to CD4^+ ^T cells [4-6]. iii) Env carbohydrates provide evasion from humoral immune responses through shielding of important protein epitopes from antibodies [7,8]. On rare occasions the carbohydrates on Env can induce rather than shield from neutralizing antibodies [9-12]. iv) Gp120-associated carbohydrates are involved in an additional means of immune evasion: the induction of immunosuppressive responses through the same interactions with C-type lectins as used by the virus during dissemination [13]. v) Gp120 glycosylation, in particular the glycosylation site within the V3 region, is involved in co-receptor use [14,15]. Collectively, alterations in gp120 Env glycosylation patterns affect several viral properties, including protein folding, (co)receptor usage, the induction of immune responses and escape from effective immune responses.

The role of individual gp120 glycans in protein structure and function is poorly understood. It is unclear which particular carbohydrates are involved in folding, C-type lectin binding, and immune evasion. A precise delineation of which sugars are important for what function is difficult because of the variation in number and location of glycosylation sites and the heterogeneous composition of the individual sugar chains. Furthermore, carbohydrates may serve different roles and multiple carbohydrates can collectively serve a single function.

In this study we have focused on one particular Env carbohydrate and investigated its role in various aspects of virus phenotype. We observed that the 386 glycan, at the base of the V4 domain, is not critical for Env folding, but its removal improved folding of an Env variant lacking the neighboring 385–418 disulfide bond, suggesting that the 386 glycan may have an auxiliary role in the presence of this disulfide bond. The 386 glycan was not essential for DC-binding and DC-mediated transmission. In contrast, the 386 carbohydrate had a major impact on neutralization sensitivity. Elimination of the 386 glycan resulted in resistance to the 2G12 antibody, but surprisingly, the contribution of this glycan appeared to be context dependent. Interestingly, all viruses lacking the 386 glycan were extremely sensitive to neutralization by the CD4BS antibody b12, suggesting that this sugar plays a role in protecting the CD4BS from antibodies.

## Results

### Evolution of a folding defective gp120

**Figure 1:**
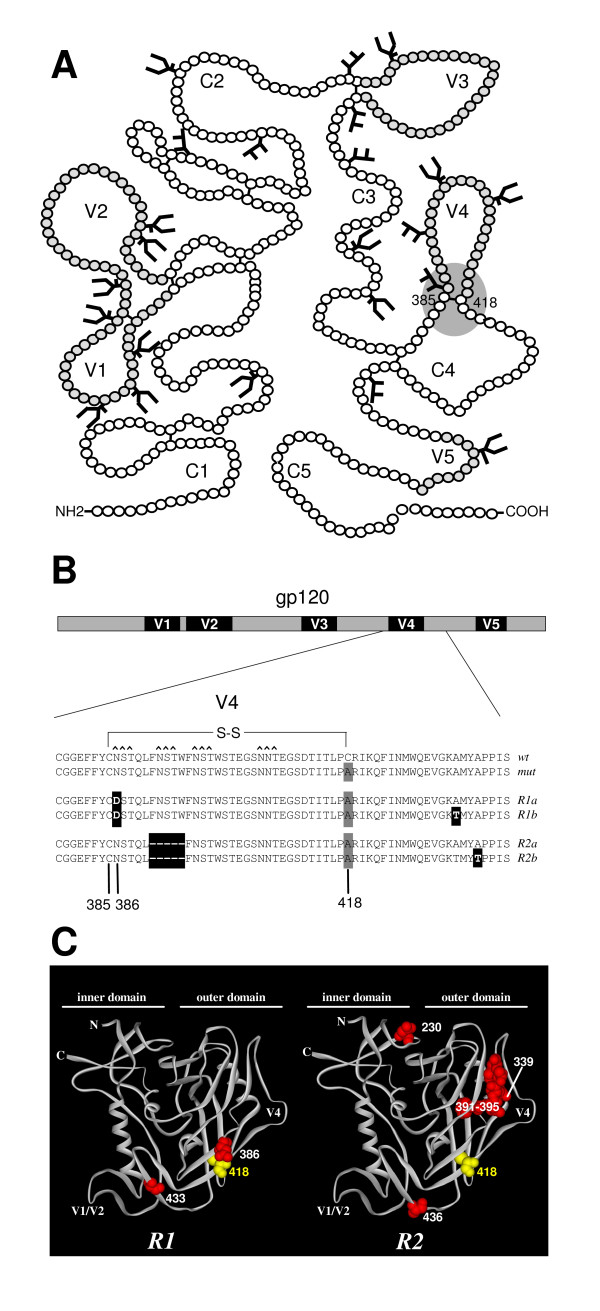
Local reversions in HIV-1 gp120. **A**. Schematic of gp120 with the 5 conserved domains (C1–C5 and five variable domains (V1–V5). The location of the V4 base disulfide bond is indicated (grey sphere). The figure is adapted from [17] and sites for N-linked glycosylation are shown. **B**. Local reversions after evolution. A detailed description of the evolution is given in materials and methods section. Sequences of the V4 loop and flanking regions of *wt*, mutant and revertant viruses. The original C418A mutation is indicated with a grey box, the reversions with black boxes. The sequences of revertant 1 are from day 39 after transfection (both 1a and 1b were derived from the day 39 sample). The sequences of revertant 2 are from day 77 (2a) and day 136 (2b) after transfection. Revertant 2 also contained reversions outside the indicated domain: T188N, N230D and R696K at day 77, and A316T, N339Y in addition to these at day 136. **C**. Locations of the reverted residues on the 3D structure of gp120. Ribbon diagram of the crystallized core of gp120 [53] with residue 418 in yellow and the reversions in red. Note that several reversions in *R2 *are not indicated because they are located outside the crystallized core (residues 188 and 316 (located in the V2 and V3, respectively), and residue 696 in gp41).

In a previous study we found that elimination of the disulfide bond at the base of V4 loop (C385–C418; fig. 1A) strongly impaired oxidative folding of HIV-1 Env [16]. However, we reproducibly observed a low level of infectivity of mutant viruses lacking this disulfide bond, although not sufficient to cause a spreading infection. A minority of the Env molecules apparently did exit the ER and reach the cell and/or virion surfaces to mediate attachment and membrane fusion. This phenotype qualified for forced protein evolution studies, with the aim of identifying and investigating escape routes that result in restoration of gp120 folding and virus replication in the absence of this particular disulfide bond. Here we describe the evolution of revertants from the C418A single mutant.

We performed multiple independent evolution experiments by transfecting the molecular clone of the HIV-1_LAI _C418A virus into SupT1 T cells followed by long-term culturing and passaging of the virus. Population sequencing revealed the sequential appearance of two amino acid substitutions: N386D and A433T (revertant *R1*, fig. 1B &1C). Sequencing of individual *env *clones revealed that several contained the individual N386D reversion alone, implying that this mutation appeared first during the course of evolution (fig. 1B). The N386D substitution disrupted an N-linked glycosylation motif (NST386-388; glycosylation site underlined) and thus led to the elimination of the oligomannose glycan that otherwise would be attached to N386 [17]. Note that this residue is located immediately adjacent to C385, the partner of C418 in the *wt *protein.

In an independent evolution culture we observed the elimination of a neighboring glycan at position 392 by deletion of the duplicate motif FNSTW (residues 391–395 or 396–400; revertant *R2*; fig 1B &1C). In addition, we found a substitution at position 436 (A436T) and some substitutions outside the V4-C4 domain (T188N, N230D, A316T, N339Y, R696K). Two of these distal changes cause the elimination of another carbohydrate (N230D, N339Y), while a third causes a putative shift of a glycosylation site by two residues (T188N; ND**T**TS to ND**N**TS; residue 188 in bold).

The defect of the C418A virus may be caused by the absence of the C385–C418 disulfide bond or the presence of a free cysteine at position 385. The unpaired cysteine at position 385 was not eliminated by the virus, suggesting that the free cysteine is not a major problem or that it is compensated for by one or more of the acquired substitutions. We also did not observe restoration of the disulfide bond by means of a first site reversion at position 418. This is probably due to the high mutational threshold to convert the introduced alanine codon back into a cysteine codon, which would require at least two nucleotide changes. In fact, we designed the mutant such that reversion to the *wt *cysteines was unlikely to occur, and thus to favor evolution of interesting second-site reversions. In summary, in two independent evolution experiments initiated with the C418A virus, a nearby carbohydrate was eliminated (*R1*: N386; *R2*: N392). Considering the importance of carbohydrates in folding, the proximity of the N386 sugar to the eliminated 385–418 disulfide bond and proximity to the CD4BS we decided to focus our subsequent experiments on *R1 *and the N386D substitution.

### Improvement of virus replication

**Figure 2:**
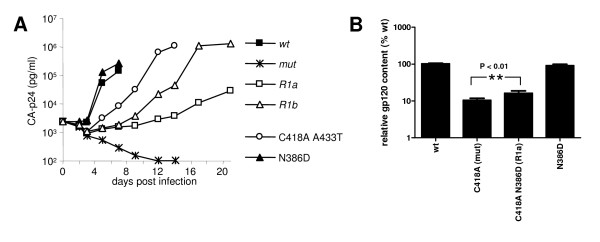
Reversions improve viral replication. **A**. 50 × 10^3^SupT1 T cells were infected with 2500 pg CA-p24 and virus spread was measured for 14 days. **B**. gp120 and CA-p24 contents in virus were measured by ELISA. The gp120 amounts were standardized for CA-p24 input and the gp120 contents of mutants in the respective fractions are given as percentages of the *wt *gp120 contents (arbitrarily set at 100). The results are representative for results from at least three independent experiments.

To establish that the substitutions we identified in *R1 *accounted for the revertant phenotype, the relevant *env *fragments were recloned into the HIV-1_LAI _(pLAI) molecular clone. Virus stocks were produced and target cells were infected with *wt*, mutant (*mut*: C418A) and revertant (*R1a*: N386D C418A, *R1b*: N386D C418A A433T) viruses. Subsequent virus spread was monitored by CA-p24 ELISA (fig. 2A). As previously described, *mut *did not cause a spreading infection [16]. *R1a *showed partial restoration of virus replication, which was further improved by the subsequent acquisition of the A433T substitution in *R1b *resulted in a further improvement of virus replication. These results indicate that a two-step evolution process took place upon removal of the 418 cysteine and, hence, the 385–418 disulfide bond, with both reversions contributing to the final revertant phenotype.

To obtain more insight in the role of the various substitutions in the restoration of virus replication, we constructed for comparison the C418A A433T double mutant. This mutant did not appear during the evolution experiment but did replicate quite efficiently, albeit with delayed kinetics compared to *wt*. We constructed the N386D single mutant to investigate the effect of this substitution and the loss of local carbohydrate on protein folding and virus phenotype. Thus, while the N386D substitution improves virus replication in the context of the C418A mutation (*R1a*), it does not appear to have a major impact on the *wt *virus.

### Restoration of gp120 content of virus particles

We previously found that folding-defective Env mutants yield virions containing virtually no Env molecules because the majority of Env is retained in the ER [16]. We studied the contribution of the N386D substitution on the relative content of Env molecules on virions, expressed as the gp120/CA-p24 ratio (fig. 2B). The gp120/CA-p24 ratio for *wt *virions was arbitrarily set at 100%. As anticipated, *mut *accumulated gp120 in the cell fraction (not shown), and very little gp120 (10.4%) was found on virus particles (fig. 2B). This result is consistent with the severe folding defect measured for this mutant. The addition of the N386D substitution in *R1a *resulted in a modest but reproducible restoration of gp120 virion content to 16.2%, suggesting that protein folding was improved by the N386D substitution. The N386D substitution caused a slightly lower gp120 content (90.2%) in the absence of C418A, indicating the improvement is specific for the C418A context.

### Partial restoration of oxidative folding

**Figure 3:**
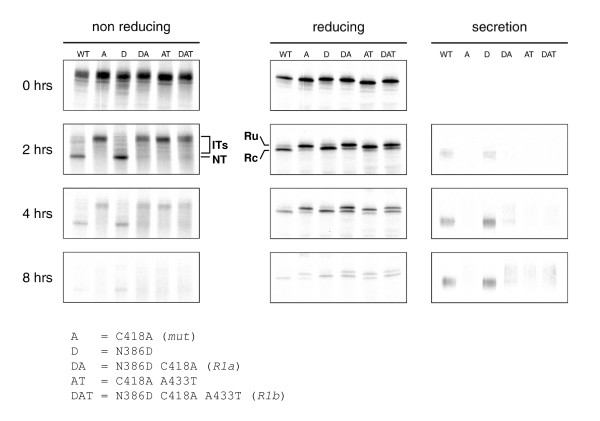
Reversions partially restore gp120 folding. HeLa cells were infected with VVT7 and transfected with plasmids encoding mutant and revertant or *wt *gp120. Cells were pulse-labeled for 5 min. and chased for the indicated times. Cells were lysed and gp120 was immunoprecipitated from lysates. Immunoprecipitates were deglycosylated with endoH and analyzed by either reducing or nonreducing 7.5% SDS-PAGE. Folding intermediates (ITs), the native form (NT), the reduced state from which the signal peptide was cleaved off (Rc) or not (Ru) are indicated. In addition, secreted gp120 was immunoprecipitated from the culture supernatant after 8 hr chase and directly analyzed by reducing SDS-PAGE.

To study whether improved protein folding of the revertants accounted for the increase in gp120 incorporation into virions, we monitored Env maturation by pulse-chase analysis. Folding of the gp120 subunit alone is very similar to that of the gp160 precursor [18]. We therefore expressed the variant gp120 molecules in HeLa cells, radiolabeled the cells and analyzed detergent cell lysates and culture supernatants for presence and folding state of gp120. We compared maturation kinetics using three read-outs that we developed before (fig. 3)[18]. First, we analyzed the formation of disulfide bonds by following mobility changes of cellular gp120 in non-reducing SDS-PAGE. Second, we monitored signal peptide cleavage in reducing SDS-PAGE. Third, we measured secretion of gp120 into the culture supernatant by reducing SDS-PAGE.

At 0 hr, all gp120 variants displayed the same mobility in the nonreducing gel, indicating that, directly after the pulse, few if any disulfide bonds had formed (fig. 3). After 2 hrs of chase, *wt *gp120 migrated faster in the non-reducing gel indicating the formation of the fully oxidized native state (NT). In contrast, most gp120 molecules of the mutant and revertants appeared as rather unfolded protein, while a minority was present as a faster migrating 'smear', representing various species of partially oxidized folding intermediates (IT). *Mut *did not display detectable levels of the native state even after 4 hrs. The revertants, however, did in part reach the native state. *R1a *and *R1b *displayed a faint native band after 2 hrs of chase.

An unusual property of Env is that it must undergo some initial oxidative folding before its signal peptide can be removed [18]. Signal peptide cleavage therefore can be used as a measure for proper gp120 folding. In reducing gels, only a single band corresponding with the preprotein form of gp120 (Reduced uncleaved = Ru) was present directly after the pulse (fig. 3). After 2 hrs of chase, however, a prominent band just below the reduced band appeared, corresponding to gp120 from which the signal peptide had been cleaved off (Reduced cleaved = Rc). After 4 hrs of chase, no uncleaved species were detectable any longer for *wt*. Signal peptide cleavage was significantly reduced for *mut*, but the *R1a *and *R1b *gp120 molecules showed partially restored cleavage, although complete processing was not accomplished. The third read-out confirmed the partial restoration of productive folding. Unlike *mut *gp120, a fraction of the revertant gp120 molecules was secreted after 8 hrs (fig. 3). Secreted *wt *gp120 appeared as compact bands, but secreted *R1a *and *R1b *gp120 displayed a smear, which may be a consequence of slower folding kinetics as prolonged retention in the ER may lead to excessive mannose trimming, resulting in more heterogeneous glycan structures [19,20]. Alternatively, a different conformation may lead to different accessibility of the carbohydrates and result in different processing. Combined, these results are consistent with the Env incorporation and virus replication experiments and confirm that virus-driven evolution resulted in a partial repair of gp120 folding by means of the N386D and A433T substitutions. In all three read-outs, the N386D single mutant was indistinguishable from *wt*, implying that the glycan at position 386 does not play an important role in *wt *gp120 folding.

### Inhibition by soluble CD4 and AMD3100

**Figure 4:**
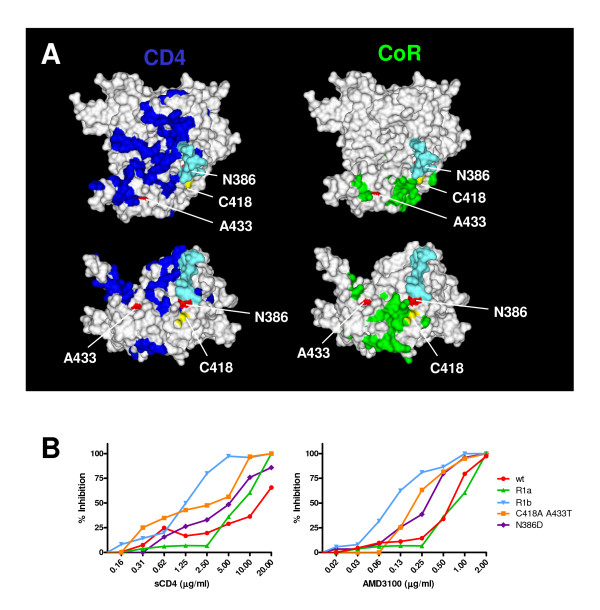
Inhibition by inhibitors of receptor interactions. **A**. Locations of mutations and reversions on the structure of gp120 relative to the receptors binding sites. In this orientation the cell membrane would be on top, the viral membrane below and allows a direct view on the CD4BS. The lower panels show a 90° rotated view, a view from the target membrane. Residue 418 is indicated in yellow, and residues 386 and 433 are indicated in red. The GlcNac2Man3 core pentose of the carbohydrate attached to N386 is indicated in cyan (modeled onto gp120 by Dr. Peter Kwong). The residues important for the interaction with CD4 and the coreceptor are indicated in blue and green, respectively. **B**. Inhibition of virus variants with sCD4 and AMD3100 on CD4^+ ^enriched lymphocytes. Inhibition curves are depicted for each of the viruses in limiting dilutions of either sCD4 (left panel) or the CXCR4 inhibitor AMD3100 (right panel).

To assess the conformation and function of the mutant and revertant Env proteins on virus particles, we studied the sensitivity of the revertant viruses to inhibitors of viral entry in a PBMC-based neutralization assay (fig. 4). The location of the binding sites of the receptor, CD4, and the CXCR4 coreceptor for HIV-1_LAI _in relation to the positions of the mutations and reversions in our viruses is given in fig. 4A. Soluble CD4 (sCD4) was used to assess the interaction of these viruses with CD4. The *wt *virus was neutralized by sCD4 with an IC_50 _of ~16 μg/ml. The various viruses (except *mut*, which did not replicate in PBMCs), were neutralized efficiently at lower concentrations of sCD4 (IC_50 _of ~1–8 μg/ml) suggesting that the affinity for CD4 is increased in these variants. *R1b *was the most sensitive to neutralization by sCD4 (IC_50 _of ~1.0 μg/ml).

As a measure for the affinity of Env for the co-receptor, which is CXCR4 in case of the HIV-1_LAI _strain, we investigated the sensitivity of the same panel of viruses to AMD3100, a small molecule inhibitor of CXCR4 (fig. 4). The *wt *virus was inhibited by AMD3100 with an IC_50 _of ~0.7 μg/ml. *R1a *was inhibited at similar concentrations, while the other virus variants were more sensitive to AMD3100 (*R1b*, N386D, C418A A433T: IC_50 _of ~0.1–0.3 μg/ml). Again *R1b *was the most sensitive (IC_50 _of ~0.1 μg/ml). The observation that less inhibitor was necessary to inhibit the interaction of these viruses with CXCR4 suggested that the affinity for CXCR4 was decreased. Taken together, the revertant *R1b *displayed increased affinity for the receptor (CD4) and decreased affinity for the co-receptor (CXCR4).

### Neutralization by antibodies b12 and 2G12

**Figure 5:**
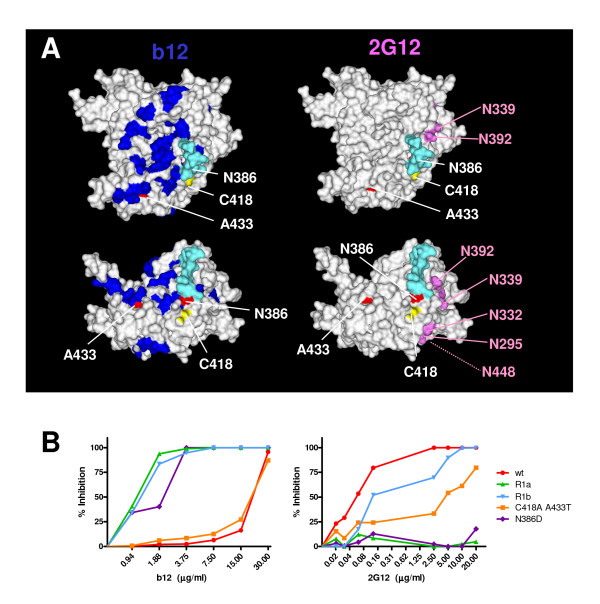
Inhibition by neutralizing antibodies. **A**. Locations of mutation and reversions on the structure of gp120 relative to the epitopes for neutralizing antibodies b12 and 2G12. Colors are the same as in fig. 4A. Residues important for b12 binding [23] are indicated in blue and the asparagines which anchor the glycans involved in 2G12 binding [10-12] are indicated in magenta. **B**. Neutralization of the virus variants with selected monoclonal antibodies on CD4^+ ^enriched lymphocytes. Neutralization curves are depicted for the various viruses in limiting dilutions of the b12 (top panel) and 2G12 (bottom panel) antibodies.

We next studied the interaction of the various viruses with the neutralizing monoclonal antibodies b12 and 2G12. The broadly neutralizing antibody b12 is directed to the CD4BS and the reversions are close to its epitope (fig. 5A) [21]. *Wt *virus was inhibited with an IC_50 _of ~20 μg/ml (fig. 5B). The C418A A433T mutant was similarly inhibited. Strikingly, all the mutants and revertants containing the N386D substitution (*R1a*, *R1b *and the N386D single mutant) were at least 10-fold more sensitive to b12 neutralization (IC_50 _of ~1.5 μg/ml), suggesting that this substitution caused an increased b12 binding.

Broadly neutralizing antibody 2G12 is directed to a number of oligomannose glycans on the outer face of gp120 (fig. 5A; [10,11]). While probably not part of the epitope itself, the carbohydrate at N386 is involved in proper formation and/or presentation of the epitope [10,11]. The *wt *virus was very sensitive to neutralization by 2G12 and the C418A A433T double mutant was slightly more resistant (IC_50 _of ~0.05 and ~4.0 μg/ml; fig. 5B). The N386D and *R1a *mutants were completely resistant at the concentrations tested, in line with studies implying the indirect involvement of the 386 glycan in 2G12 binding [10-12]. Strikingly, *R1b*, which also lacks the 386 glycan, was sensitive to 2G12 neutralization.

### Effect of the N386D substitution on neutralization

**Figure 6:**
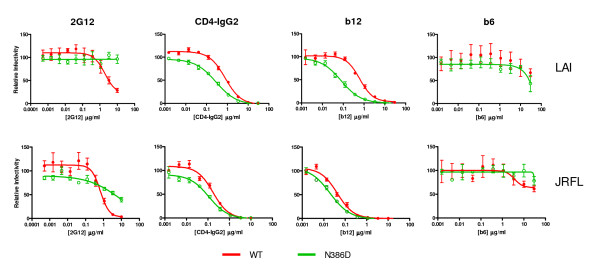
Neutralization in single cycle infection assays. *Wt *an d N386D HIV-1_LAI _virus and *wt *and N386D pseudovirus derived from HIV-1_JR-FL _were preincubated with antibody and subsequently incubated with TZM-bl cells containing a luciferase reporter construct under control of the HIV-1 LTR as described in the materials and methods section. The luciferase activity in the absence of inhibiting reagents was set at 100%.

To further characterize the contribution of N386 to neutralization and to corroborate the data obtained in the PBMC-based neutralization experiments, we performed single cycle neutralization experiments using complete HIV-1_LAI _virus (fig. 6 and table 1). We also included pseudovirus of the CCR5-using HIV-1_JR-FL _strain. As in the PBMC-based assay, the N386D mutant was completely resistant to 2G12 neutralization while the *wt *virus was sensitive (IC_50 _of >10 μg/ml and 1.93 μg/ml, respectively; fig. 6 and table 1). Similar results were obtained using the HIV-1_JR-FL _strain, but the HIV-1_JR-FL _N386D variant was not as resistant to 2G12 neutralization as the HIV-1_LAI _variant (IC_50_s of 0.66 μg/ml (*wt*) and 7.96 μg/ml (N386D)), confirming that the involvement of the 386 glycan to the 2G12 epitope is variable and/or context dependent.

**Table 1:** Neutralization in single cycle assays (50% inhibitory concentrations (μg/ml))^a^

	LAI (whole virus)		JR-FL (pseudovirus)	
	*wt*	N386D	*wt*	N386D

2G12	1.93	>10	0.66	7.96
CD4-IgG2	0.78	0.28	0.17	0.11
b12	0.62	0.079	0.043	0.020
b6	>10	>10	>10	>10

^a ^The IC_50 _values were derived from the experiment in fig. 6 as described in the materials and methods section.

We next tested the accessibility of the CD4BS. The HIV-1_LAI _N386D variant was approximately 3-fold more sensitive to CD4-IgG2 neutralization compared to *wt *(IC_50 _of 0.28 μg/ml and 0.78 μg/ml, respectively), mimicking the results obtained in the PBMC-based neutralization experiments using sCD4. Similar results were obtained using HIV-1_JR-FL _pseudovirus, although the difference in IC_50 _was less than 2-fold (0.11 μg/ml versus 0.17 μg/ml). Both wt (pseudo)viruses were sensitive to b12 neutralization, but the N386D variants were much more sensitive (8-fold for HIV-1_LAI _and 2-fold for HIV-1_JR-FL_). Apparently the role of N386 in protection of the CD4BS is less pronounced in HIV-1_JR-FL _Env. Monoclonal antibody b6 is derived from the same phage library as b12 [22], however, it does not neutralize very efficiently, presumably because its angle of binding to gp120 is incompatible with binding to the Env trimer [23]. We indeed observed that *wt *HIV-1_LAI _and *wt *HIV-1_JR-FL _were both resistant to b6 neutralization. Furthermore, the N386D substitution did not increase the sensitivity of these viruses to b6. These data indicate the N386D specifically increases exposure of the neutralizing b12 epitope on the functional Env trimer, but not of the nonneutralizing b6 epitope. This is in contrast to HIV-1_LAI _viruses lacking the V1/V2 domain which show increased sensitivity to both b12 and b6 (R.W. Sanders *et al*. unpublished results). Allthough these data should be extended by testing other nonneutralizing CD4BS antibodies, the specific improvement of exposure of the b12 epitope may be relevant to vaccine design aimed at inducing b12-like antibodies, while avoiding the elicitation of nonneutralizing antibodies.

### DC binding and transmission

**Figure 7:**
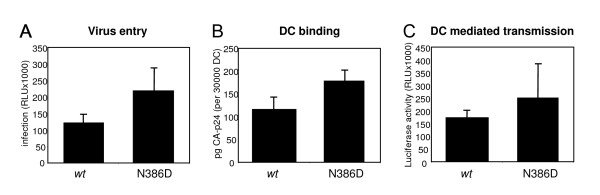
The N386 carbohydrate is not essential for DC-mediated HIV-1 transmission. **A**. LuSIV cells were incubated with the two viruses and luciferase was measured after 24 hours. RLU, relative light units. Error bars represent standard deviations. **B**. DCs were incubated for 2 hours with *wt *or N386D virus, followed by extensive washing to remove unbound virus. Viral capture was subsequently determined by lysis of the cells and CA-p24 ELISA. **C**. DCs were incubated for 2 hours with *wt *or N386D virus, followed by extensive washing to remove unbound virus. DCs were subsequently cocultured with LuSIV cells to allow HIV-1 transmission. Transmission efficiency was determined by measuring luciferase activity after 24 hours.

Because oligomannose containing carbohydrates on gp120 interact with C-type lectins on dendritic cells (DC) and facilitate DC-mediated transmission to T cells [4-6], we examined whether the N386 glycan, which is thought to exist as an oligomannose carbohydrate on gp120 [17], contributed to binding of HIV-1 to DC. First, we determined the infectivity of both *wt *and N386D HIV-1_LAI _in a single-cycle infection assay using LuSIV reporter cells. These reporter cells contain the firefly luciferase gene downstream of the LTR promoter, resulting in Tat-mediated luciferase expression, which is a measure of infectivity [24]. In accordance with the replication experiments, we found no significant differences (fig. 7A). We next incubated DC with both viruses for 2 hrs, followed by washing steps to remove unbound virus. After lysis of the cells, we measured the amount of captured HIV by CA-p24 ELISA and found no significant difference in *wt *or N386D virus capture by DC (fig. 7A). These results show that the N386 glycan is not essential for binding to DC. Finally, we tested whether *wt *or N386D virus was transmitted with equal efficiency by DC to T cells. DC were incubated with virus for 2 hrs, followed by washing steps and addition of LuSIV cells. Since the LuSIV cells are harvested within 24 hrs for luciferase measurement, there is no significant T cell spread of newly produced HIV-1 virions, such that luciferase activity is a quantitative measure of the amount of virus that is transmitted by DC. We found no significant differences in transmission efficiency (fig. 7C), which was expected since both capture by DC and infectivity of N386D was similar to wt HIV-1 (figs. 7A and 7B). Since HIV-1 binding to monocyte-derived DC predominantly takes place via C-type lectins such as DC-SIGN [6,25-27], these results imply that the individual 386 carbohydrate is not essential for C-type lectin binding and DC-mediated transmission.

## Discussion

The carbohydrate component of Env, comprising ~50% of its molecular weight, is much less well characterized than the protein component. The sugars facilitate different functions and display an unusual plasticity in terms of composition, structure and location. Thus, sugar chains can move around the molecule, they are flexible and their composition depends on the local environment. Furthermore, the carbohydrates on Env can serve many purposes. This study on the 386 carbohydrate underlines some of these features.

The N386D substitution does not appreciably affect oxidative folding of gp120, but it does improve folding of a gp120 variant lacking the 385–418 disulfide bond caused by the C418A substitution. Perhaps the 386 sugar has a supporting role in folding of *wt *gp120 that was not apparent in our assays, but may be more prominent in (resting) primary immune cells, which may enforce more constraints on protein folding than transformed cell lines. We observed the loss of a nearby glycan in two independent evolution experiments using the C418A single mutant. The carbohydrates at positions 386 and 392 were not lost in evolution studies using the C385A C418A double mutant [16]. It is therefore possible that the loss of local carbohydrate somehow compensates for the presence of an unpaired cysteine at position 385.

Although the 386 glycan exists as an oligomannose carbohydrate that could potentially bind to DC-SIGN and/or other lectins on DC [17], our DC transmission studies suggest that N386 is not essential for gp120 binding to DC. However, recent studies show that although the binding of DC-SIGN to gp120 has some specificity it is also considerably promiscuous [28]. It remains possible that N386 plays a facilitating role in binding to DC-SIGN and/or other lectins *in vivo*, in combination with other carbohydrates on gp120. A better definition of the binding domain(s) for lectins such as DC-SIGN on gp120 is warranted.

The 386 carbohydrate was previously found to be involved in 2G12 binding, such that it facilitates the proper formation or presentation of the 2G12 mannose epitope [10,11]. Although not directly part of the 2G12 epitope, the removal of N386 may result in different modification of the neighbouring sugars that form the core epitope. 2G12 requires terminal mannose residues on oligomannose chains. The removal of the 386 sugar may result in enhanced accessibility of the sugars that normally bind 2G12, resulting in processing of the oligomannose chains to complex carbohydrate and consequently a loss of terminal mannose residues. Our findings confirm these results, but also show that the contribution of the 386 carbohydrate is context dependent. Thus, in the presence of the C418A and A433T mutations the 386 sugar is not critical for formation or presentation of the 2G12 epitope. Apparently, these mutations result in decreased accessibility of the 2G12 carbohydrates even in the absence of the 386 sugar.

Much effort is put into attempts to elicit antibodies similar to the few broadly neutralizing antibodies that have been isolated and characterized (b12, 2G12, 4E10, 2F5). The b12 epitope/CD4BS is an attractive target since most, if not all, primary HIV-1 viruses need CD4 for entry. We show here that the removal of the 386 glycan significantly improves b12 and CD4 binding to virus-associated Env. A recent study showed that a N386Q substitution in a primary subtype C isolate also significantly enhanced the sensitivity to b12 neutralization, strengthening the notion that the 386 carbohydrate hides the b12 epitope [29]. Similar results were obtained with an N386A substitution in JR-FL (R. Pantophlet and D. Burton, personal communication). These data also indicate that it is probably not the chemical property of the introduced amino acid that causes the increased sensitivity, but rather the lack of the carbohydrate. Interestingly, both the asparagine at position 386 and the attached glycan are contact sites for b12 on monomeric gp120 [30], but apparently, in the context of the functional trimer, the 386 glycan located on the edge of gp120's silent face shields the CD4BS and the b12 epitope [7].

Since the 386 carbohydrate is involved in protection of the CD4BS from antibodies it may be worthwhile to consider the elimination of this carbohydrate in Env-based immunogens. Several studies demonstrated a benefit from the reduction of carbohydrates in terms of the exposure of the CD4BS and the induction of neutralizing antibodies, although other studies did not show such an effect [31-39]. However, systematic studies analyzing the contribution of every single carbohydrate are lacking. Furthermore, the antigen scaffold used in these studies (virus-associated Env, monomeric gp120 and incompletely cleaved or uncleaved gp140/gp150/gp160) may not be optimal for such an analysis. The contribution of sugar chains to protection from antibody recognition may be quite different in the context of a better mimic of the functional trimer [7].

## Conclusion

Although the 386 carbohydrate may play a role in Env folding, this is not a crucial one, underlined by the fact that it is not absolutely conserved among primary isolates (15% variability in clades A, B, and C [10]). Despite the observation that this sugar is present as an oligomannose carbohydrate [17], it does not seem to play an essential role in the binding to DC and DC-mediated transmission. As previously reported, the 386 sugar contributes to 2G12 binding, but we show here that this contribution is not direct. The main finding of our study is that the glycan at residue 386 plays a role in the protection of the CD4BS against antibody recognition. Although antigenicity cannot be directly correlated with immunogenicity, removal of the 386 glycan in trimeric Env vaccines may facilitate elicitation of neutralizing b12-like CD4BS antibodies. Studies to test this hypothesis are in progress.

## Methods

### Cloning

The pRS1, pcDNA3-Env-gp120 and pLAI plasmids containing the appropriate mutations in the *env *gene were generated as described previously [16]. PCR-generated gp120 sequences from evolved viruses (see below) were cloned into the pRS1 shuttle vector [40] using the *Bsa*B1 and *Nhe*1 sites and subsequently cloned into the pLAI infectious molecular clone [41] as *Sal*I-*Bam*HI fragments. *Not*I-*Xho*I fragments were subcloned into the pcDNA3 expression vector for use in folding experiments. Numbering of individual amino acids is based on the sequence of HXB2 gp160.

### Cells and transfections

HeLa cells (ATCC) and HT1080 cells were cultured in MEM (Life technologies) supplemented with 10% FCS (Hybond), penicillin (100 U/ml; Sigma-Aldrich), streptomycin (100 μg/ml; Invitrogen). SupT1 cells were cultured in RPMI medium 1640 (Life Technologies) supplemented with 10% FCS, penicillin and streptomycin. C33A cervix carcinoma cells were maintained in DMEM (Life Technologies), supplemented with 10% FCS, penicillin and streptomycin, as previously described [42]. SupT1 and C33A cells were transfected with pLAI by electroporation and Ca_3_(PO_4_)_2 _precipitation, respectively, as described previously [43]. The reporter cell line LuSIV was kindly donated by Janice E. Clements (Johns Hopkins University School of Medicine, Baltimore, MD). This CEMx174-derived cell line contains the firefly luciferase reporter gene downstream of the SIV_mac_239 LTR. Infection by HIV-1 results in Tat-mediated expression of luciferase, which can be measured 24 hours later. Cells were maintained in RPMI medium, supplemented with 10% FCS, 2 mM sodium pyruvate, 10 mM HEPES, 2 mM L-glutamine, penicillin (100 U/ml), streptomycin (100 μg/ml), and hygromycin B (300 μg/ml) to maintain the luciferase construct. For experiments with DCs, hygromycin B-free medium was used. Peripheral blood mononuclear cells (PBMCs were isolated from fresh buffy coats (Central Laboratory Blood Bank, Amsterdam) by standard Ficoll-Hypaque density centrifugation. PBMCs were frozen in multiple vials at a high concentration and, when required, thawed and activated with 5 μg/ml phytohemagglutinin (Sigma) and cultured in RPMI medium containing 10% FCS, penicillin (100 U/ml), streptomycin (100 μg/ml), and recombinant interleukin-2 (rIL-2) (100 units/ml). On day 4 of culture, the cells underwent CD4^+ ^enrichment by incubating the PBMC with CD8 immunomagnetic beads (Dynal) and separating out the CD8^+ ^lymphocytes.

### Viruses and infections

Virus stocks were produced by transfecting C33A cells with the appropriate pLAI constructs. The virus containing supernatant was harvested 3 days post-transfection, filtered and stored at -80°C and the virus concentration was quantitated by capsid CA-p24 ELISA as described previously [44]. These values were used to normalize the amount of virus in subsequent infection experiments. Infection experiments were performed as follows. 50 × 10^3 ^SupT1 T cells were infected with 2500 pg CA-p24 of C33A-produced HIV-1_LAI _per well in a 96-well plate, and virus spread was measured for 14 days using CA-p24 ELISA. For single-cycle experiments to determine viral infectivity, 40 × 10^3 ^LuSIV cells/200 μl/well were infected virus (2 ng CA-p24/well), followed by luciferase measurement after 24 hrs, as described previously [45]. JR-FL pseudoviruses were produced by cotransfection of 3.3 μg pSV7D-JR-FL gp160 (a gift from James Binley) and 1,7 μg pSG3-ΔEnv (obtained through the AIDS Research and Reference Reagent Program, Division of AIDS, NIAID, NIH from Drs. John C. Kappes and Xiaoyun Wu) into 293T cells.

### Virus evolution

Evolution experiments were essentially performed as previously described [40,46]. 5 × 10^6 ^SupT1 cells were transfected with 10 μg of the pLAI C385A/C418A construct by electroporation. The culture was inspected regularly for the emergence of revertant viruses, using CA-p24 ELISA and/or the appearance of syncytia as indicators of virus replication. Cells were passaged twice a week. Decreasing amounts (1.0 ml, 100 μl, 10 μl, 1.0 μl) of virus were passaged cell free onto fresh cells when the cells were (almost) wasted due to infection. The intervals and volumes of cell free passage depended on the replication efficiency and cytopathogenicity of the evolving virus. Upon identification of a faster replicating virus, DNA was extracted from infected cells [47] and proviral gp120 sequences were PCR-amplified with primers A (5'-GCTCCATGGCTTAGGGCAACATATATCTATG-3') and B (5'-GTCTCGAGATGCTGCTCC-3') and sequenced. Population sequencing revealed two reversions: N386D and A433T (fig. 1). Half of the individual *env *clones that were sequenced contained these two substitutions. However, half of the clones only had the individual N386D reversion, implying that this mutation appeared first during the course of evolution (fig. 1).

### HIV-1 neutralization assay using primary CD4^+ ^lymphocytes

Viruses were tested for their relative inhibition sensitivity against increasing concentrations of either sCD4 (ImmunoDiagnostics, Inc.) or the CXCR4 binding compound AMD3100 (a generous gift from Dr D Schols). TCID_50 _values were determined on purified CD4^+ ^lymphocytes isolated from an individual who did not carry the Δ32CCR5 allele (CCR5^+/+^) screened for by standard PCR. CD4^+^-enriched lymphocytes were plated at 2 × 10^5 ^cells/well in 96-well plates with 5-fold serial dilutions of the virus. The cells were fed on day 7 with fresh media and scored on day 14 for p24 levels, with the number of positive wells being used to identify the TCID_50 _value for each virus. Each virus (100 TCID_50 _in 50 μl of the culture RPMI medium) was mixed with an equal volume of serially diluted compound for 1 hr at 37°C in flat-bottomed 96-well plates and all neutralization reactions were performed in triplicate. After 1 hour of incubation 2.0 × 10^5^positively selected CD4^+ ^lymphocytes were added to each well in 100 μl of culture media containing rIL-2. For each neutralization experiment a positive control, virus incubated with cells in the absence of inhibitory compound, and a negative control, virus in the absence of cells, was included. The negative control p24 concentration was subtracted from all test results. HIV-1 specific p24 production was measured on day 7, 10 and 14 of culture. On these days half the cell supernatant of each well was replaced with fresh medium. The day chosen for calculating neutralizing responses was based on the day the p24 value of the positive control well peaked and percentage neutralization was calculated by determining the reduction in p24 production in the presence of the agent compared to that for the cultures with virus only.

### Single cycle virus Neutralization

The TZM-bl reporter cell line [48,49] stably expresses high levels of CD4 and HIV-1 co-receptors CCR5 and CXCR4 and contains the luciferase and β-galactosidase genes under control of the HIV-1 LTR promoter. The TZM-bl cell line was obtained through the NIH AIDS Research and Reference Reagent Program, Division of AIDS, NIAID, NIH: TZM-bl from Dr. John C. Kappes, Dr. Xiaoyun Wu and Tranzyme Inc. One day prior to infection, TZM-bl cells were plated on a 96-wells plate in DMEM medium (Gibco) containing 10% fetal bovine serum, 1× MEM (Gibco) and penicillin/streptomycin (both at 100 units/ml) and incubated at 37°C with 5% CO_2_. A fixed amount of HIV-1_LAI _virus produced in C33A cells (500 pg CA-p24) or HIV-1_JR-FL _pseudovirus produced in 293T cells (500 pg CA-p24) was pre-incubated for 30 min at room temperature with escalating concentrations of monoclonal antibodies (2G12: obtained from Hermann Katinger through the NIH AIDS Research and Reference Reagent Program; b6 and b12: gifts from Dennis Burton) or CD4 (sCD4, CD4-IgG2: gifts from William Olson, Progenics Pharmaceuticals). This mixture was added to the cells in the presence of 400 nM saquinavir (Roche) and 40 μg/ml DEAE in a total volume of 200 μl. Two days post-infection the medium was removed and cells were washed once with PBS and lysed in Reporter Lysis buffer (Promega). Luciferase activity was measured using the Luciferase Assay kit (Promega) and a Glomax luminometer according to the manufacturer's instructions (Turner BioSystems). All infections were performed in duplicate and luciferase measurements were also performed in duplicate. Uninfected cells were used to correct for background luciferase activity. The infectivity of each mutant without inhibitor was set at 100%. Non-linear regression curves were determined and IC_50 _values were calculated using Prism software version 4.0c.

### Generation of monocyte-derived dendritic cells

Monocyte-derived dendritic cells were genereated as previously described [42,45,50]. Peripheral blood mononuclear cells (PBMC) were isolated by density centrifugation on Lymphoprep (Nycomed). Subsequently, PBMC were layered on a Percoll gradient (Pharmacia) with three density layers (1.076, 1.059, and 1.045 g/ml). The light fraction with predominantly monocytes was collected, washed, and seeded in 24-well culture plates (Costar) at a density of 5 × 10^5 ^cells per well. After 60 min at 37°C, nonadherent cells were removed, and adherent cells were cultured to obtain iDC in Iscove's modified Dulbecco's medium (IMDM; Life Technologies Ltd.) with gentamicin (86 μg/ml; Duchefa) and 10% fetal clone serum (HyClone) and supplemented with GM-CSF (500 U/ml; Schering-Plough) and IL-4 (250 U/ml; Strathmann Biotec AG). At day 3, the culture medium with supplements was refreshed. At day 6, maturation was induced by culturing the cells with poly (I:C) (20 μg/ml; Sigma-Aldrich). After two days, mature CD14^- ^CD1b^+ ^CD83^+ ^DC were obtained. All subsequent tests were performed after harvesting and extensive washing of the cells to remove all factors.

### DC-binding experiments

Fully matured DC were incubated in a 96-well-plate (40 × 10^3 ^DC/100 μl/well) with virus (5 ng CA-p24/well) for 2 hr at 37°C. After centrifugation at 400 × g, the DC were washed with PBS to remove unbound virus. This step was repeated twice and was followed by lysis of the cells and CA-p24 ELISA to determine the amount of HIV-1 captured by the DC.

### Single-cycle DC transmission assay

Fully matured DC were incubated in a 96-well-plate (40 × 10^3 ^DC/100 μl/well) with virus (5 ng CA-p24/well) for 2 hr at 37°C. The DC were washed with PBS after centrifugation at 400 × g to remove unbound virus. Washing was repeated twice, followed by addition of 40 × 10^3 ^LuSIV cells. After 24 hr, LuSIV cells were harvested for luciferase measurement. 40 × 10^3 ^LuSIV cells grown without DC or HIV-1 were used to obtain the background luciferase value, which was subtracted from all data.

### Quantitation of gp120 in virus fractions

C33A cells were transfected with 40 μg pLAI per T75 flask. Medium was refreshed at day one post-transfection. The culture supernatant was harvested at 3 days post-transfection, centrifuged and passed through a 0.45 μm filter to remove residual cells and debris. Cells were resuspended in 1.0 ml lysis buffer (50 mM Tris (pH 7.4) 10 mM EDTA, 100 mM NaCl, 1% SDS). Virus particles were pelleted by ultracentrifugation (100,000 *g *for 45 min at 4°C) and resuspended in 0.5 ml lysis buffer. The virus free supernatant, containing gp120 shed from the cell and virion surface, was concentrated using Amicon centrifugal filter units (Millipore) and SDS was added to a 1% final concentration.

Gp120 in cell, virion and supernatant fractions was measured as described previously [10,51], with minor modifications. ELISA plates were coated overnight with sheep antibody D7324 (10 μg/ml; Aalto Bioreagents), directed to the gp120 C5 region, in 0.1 M NaHCO_3_. After blocking with 2% milk powder in Tris-buffered saline (TBS) for 30 min, gp120 was captured by incubation for 2 hr at room temperature. Recombinant HIV-1_LAI _gp120 (Progenics Pharmaceuticals) was used as a reference. Unbound gp120 was washed away with TBS and purified serum Ig from an HIV-1 positive individual (HIVIg) was added for 1.5 hr in 2% milk, 20% sheep serum, 0.5% Tween-20. HIVIg binding was detected with alkaline phosphatase conjugated goat anti-human Fc (1:10,000, Jackson Immunoresearch) in 2% milk, 20% sheep serum, 0.5% Tween-20. Detection of alkaline phosphatase activity was performed using AMPAK reagents (DAKO). The measured gp120 contents in cells, virus and supernatant were normalized for CA-p24.

### Env folding

For folding assays, mutant gp120 was expressed using a recombinant Vaccinia virus vector system. Folding of gp120 mutants was analyzed by pulse-chase labeling and immunoprecipition with rabbit sera raised against gp160 from lysed cells and recognizing all forms of gp160, as described [18]. *Wt*, mutant and revertant gp120 were expressed in HeLa cells under control of the T7 promoter using a recombinant Vaccinia virus vector system [52]. Cells were placed on ice directly after the pulse or after various chase times. Culture supernatants were collected and cells were washed, incubated with iodoacetamide to block free sulfhydryl groups and lysed. gp120 from cell lysates and culture supernatants was immunoprecipitated and treated with Endoglycosidase H to remove oligomannose glycans. This results in deglycosylation of virtually all intracellular gp120 since the contribution of EndoH-resistant gp120 in the cell is negligible [18]. Formation of disulfide bonds was assayed by SDS-PAGE mobility changes of deglycosylated, alkylated, non-reduced samples. Reduced samples were used to follow signal sequence cleavage.

## Competing interests

The author(s) declare that they have no competing interests.

## Authors' contributions

RWS performed the evolution studies and drafted the manuscript. EvA and IML performed the folding assays, AN carried out the PBMC-based neutralization experiments and FG performed the DC-transmission experiments. IljaB, DE, MM, EB and MMD carried out the cloning, virus replication experiments, neutralization experiments and gp120 ELISAs. RWS, InekeB, BB and WAP conceived and supervised the study. All authors read and approved the final manuscript.
